# Association of Sensory Impairments With Cognitive Decline and Depression Among Older Adults in China

**DOI:** 10.1001/jamanetworkopen.2020.14186

**Published:** 2020-09-29

**Authors:** Hongguo Rong, Xiaozhen Lai, Rize Jing, Xiao Wang, Hai Fang, Elham Mahmoudi

**Affiliations:** 1 China Center for Health Development Studies, Peking University, Beijing, China; 2 Institute for Excellence in Evidence-Based Chinese Medicine, Beijing University of Chinese Medicine, Beijing, China; 3 Peking University School of Public Health, Beijing, China; 4 Beijing Key Laboratory of Mental Disorders, The National Clinical Research Center for Mental Disorders, Anding Hospital, Capital Medical University, Beijing, China; 5 Peking University Health Science Center, Chinese Center for Disease Control and Prevention Joint Center for Vaccine Economics, Beijing, China; 6 Key Laboratory of Reproductive Health, National Health Commission of the People’s Republic of China, Beijing, China; 7 Department of Family Medicine, University of Michigan, Ann Arbor

## Abstract

**Importance:**

Sensory impairments may heighten the risk of premature brain aging. Little is known regarding the association of sensory impairments with cognitive function and depression in China.

**Objective:**

To examine the association of visual and/or hearing impairments with cognitive decline and depression.

**Design, Setting, and Participants:**

This cross-sectional study used the data from 18 038 respondents to the 2015 survey of the China Health and Retirement Longitudinal Study, a nationally representative survey of adults aged 45 years or older and their spouses.

**Exposures:**

The presence of sensory impairments was identified by self-reported assessment of visual and/or hearing functions.

**Main Outcomes and Measures:**

Overall, 3 composite measures were used to assess the respondents’ cognitive performance, including episodic memory, mental intactness, and global cognitive function. Depression was examined using the 10-item Center for Epidemiological Studies–Depression scale score. Multiple generalized linear regression models, adjusting for self-reported sociodemographic characteristics, health behaviors, chronic conditions, and participants’ overall health, were used.

**Results:**

Of 18 038 respondents, 9244 (51.2%) were women. The mean (SD) age was 59.9 (9.7) years. Respondents with visual impairment had poorer episodic memory (β = –0.12; 95% CI, –0.19 to –0.05) and global cognition (β = –0.16; 95% CI, –0.31 to –0.02) and a greater risk of depression (odds ratio, 1.78; 95% CI, 1.59 to 1.99) than those without visual impairment. Respondents with hearing impairment had poorer episodic memory (β = –0.24; 95% CI, –0.30 to –0.18), mental intactness (β = –0.19; 95% CI, –0.28 to –0.10), and global cognition (β = –0.43; 95% CI, –0.55 to –0.31) as well as a greater risk of depression (odds ratio, 1.57; 95% CI, 1.44 to 1.70) than those without hearing impairment. People who reported dual sensory impairment performed worse on all of the aforementioned outcome measures (episodic memory: β = –0.23; 95% CI, –0.31 to –0.14; mental intactness: β = –0.13; 95% CI, –0.27 to –0.0003; global cognition: β = –0.37; 95% CI, –0.55 to –0.19; depression: odds ratio, 2.19; 95% CI, 1.90 to 2.52).

**Conclusions and Relevance:**

In this study, visual and hearing impairments were independently and together associated with poorer cognitive and depression outcomes. A more comprehensive and integrated system of care, covering vision, hearing, and cognition, is needed in China’s health care system to address age-related sensory impairments.

## Key Points

**Question:**

Are visual and/or hearing impairments associated with cognitive decline and depression?

**Findings:**

In this cross-sectional study of 18 038 participants aged 45 years or older in China, visual impairment was associated with worse performance in episodic memory and global cognition as well as with worse depression symptoms. Hearing impairment was also associated with poorer performance in episodic memory, mental intactness, and global cognition.

**Meaning:**

In this study, visual and/or hearing impairments were associated with higher risks of cognitive decline and depression among middle-aged and older adults in China.

## Teaser

This cross-sectional study examines the association of visual and/or hearing impairments with cognitive decline and depression among older adults in China.

## Introduction

The risk of visual and hearing impairments increases with age, and some emerging literature considers them risk factors for Alzheimer disease and related dementia.^1^ By 2050, it is estimated that more than 900 million people in the world will be living with clinically meaningful hearing loss.^2^ It is also estimated that approximately 2.2 billion people in the world experience some form of vision impairment, including blindness and moderate to severe vision impairment.^3^

Visual and hearing impairments are chronic conditions that may increase the risk of cognitive decline and depressive symptoms.^4,5,6^ Although the prevalence of visual impairment increases with age,^7^ some vision impairments can be corrected via wearing glasses or surgery.^8^ Clinical evidence suggests that cataract surgery resulting in improved vision is associated with improved cognitive performance and increased gray matter volume in the cortex.^9^ Similarly, hearing impairment is increasingly common among older adults. Despite evidence showing that hearing loss increases the risk of cognitive impairment and that it is somewhat modifiable, in most cases, hearing loss remains untreated.^10,11^ It has been shown that older adults with peripheral hearing loss have a 24% higher risk of mild cognitive impairment than those without.^12^

The number of older adults with visual and/or hearing loss is growing worldwide, and China, with a rapidly aging population, is no exception. Compared with Europe and the United States, China has a substantially higher prevalence of sensory impairments, and research regarding the association of sensory impairments with cognitive decline and depression is lacking.^13^ Using a nationally representative sample of adults aged 45 years and older, we aimed to explore the association of sensory impairments with cognitive function and depression in China. We hypothesized that sensory impairments are strongly associated with cognitive decline and depressive symptoms.

## Methods

### Data and Study Sample

**Figure 1.**
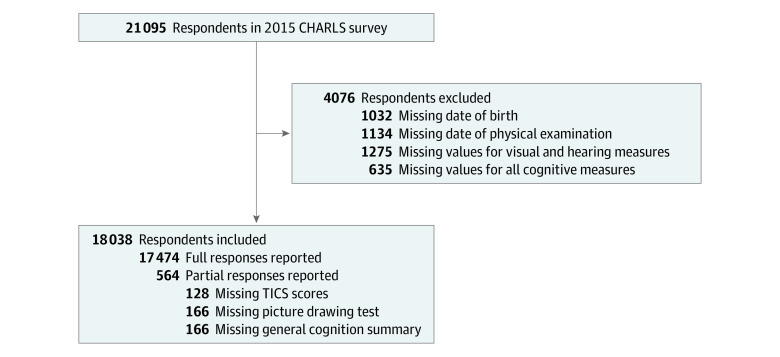
Flow Chart of the Study Sample From the 2015 China Health and Retirement Longitudinal Study (CHARLS) TICS indicates Telephone Interview of Cognitive Status.

The China Health and Retirement Longitudinal Study (CHARLS) contains a nationally representative sample of Chinese adults aged 45 years or older and their spouses. The first wave of CHARLS was collected in 2011, with follow-up waves every 2 years. The national baseline survey of CHARLS included 17 708 respondents in 150 counties or districts and 450 villages or urban communities throughout the country. We used the latest CHARLS wave (2015) with 21 095 individuals to investigate the association of self-assessed sensory impairments with cognitive functions and depression. Figure 1 shows the schematic flow of the study sample. Our final sample included 18 038 individuals with and without sensory impairments.

The CHARLS program protocol complied with the Declaration of Helsinki^14^ and was ethically approved by the Peking University institutional review board.^15,16^ The present study involved a secondary analysis of established data sets and was not subject to ethical approval according to the London School of Economics and Political Science research ethics policy and procedures. This cross-sectional study obtained written informed consent from all participants and was conducted following the Strengthening the Reporting of Observational Studies in Epidemiology (STROBE) reporting guideline.^17^

### Cognitive Function and Depression

In line with the Health and Retirement Study^18,19^ and other studies on cognitive functions,^20,21^ 3 composite measures were used to access the respondents’ cognitive performance: a word recall test, Telephone Interview of Cognitive Status (TICS-10), and a pentagon drawing test.^22^ The word recall test was used to assess episodic memory. In the recall test, respondents were asked to immediately repeat 10 Chinese nouns that had just been read to them in any order (immediate recall) and recall the same word list 4 minutes later (delayed recall). The episodic memory score was calculated by finding the mean of the immediate and delayed recalls, ranging from 0 to 10.^23^ The TICS-10 and a picture drawing test were used to evaluate mental intactness^24^ in terms of time orientation, numerical ability, and visuospatial ability. Participants were asked to complete 7 serial subtractions from 100 (up to 5 times); answer what day it was (month, day, year), what day of the week it was, and what season of the year it was; and redraw a picture of 2 overlapped pentagons. Their scores from the questions were aggregated into a single mental intactness score, which ranged from 0 to 11.^22^ The global cognitive function was the summation of the episodic memory and mental intactness scores. For all these measures, higher scores indicate better cognitive function.

Furthermore, CHARLS uses the 10-item Center for Epidemiological Studies–Depression scale (CES-D 10) to assess depression symptoms.^25^ As a brief screening tool for depressive symptoms, the CES-D 10 was validated among middle-aged and older respondents in China.^26,27^ The CES-D 10 includes 10 items with 4 answers on a 4-scale metric, ranging from rarely or none of the time (<1 day), some days (1-2 days), occasionally (3-4 days), or most of the time (5-7 days). Each item was scored from 0 to 3, with a total possible score ranging from 0 to 30 (Cronbach α = 0.815).^25^ A higher score reflects more severe depressive symptoms. Previous studies suggested a cutoff point greater than or equal to 10 to differentiate between those with and without depression.^15,28^

### Sensory Function

The presence of sensory impairment was identified by self-reported assessment of visual and hearing functions. Similar to the Survey of Health, Aging and Retirement in Europe, CHARLS collected self-reported data on visual functions using 2 questions: “Is your eyesight for seeing things at a distance excellent (1), very good (2), good (3), fair (4), or poor (5)?” and “How good is your eyesight for seeing things up close, like reading ordinary newspaper print? Would you say your eyesight for seeing things up close is excellent (1), very good (2), good (3), fair (4), or poor (5)?” Data on hearing functions were also collected using the question: “Is your hearing excellent (1), very good (2), good (3), fair (4), or poor (5)?”

We identified respondents as having visual or hearing impairment if they reported fair or poor vision (for either long distance or near vision) or hearing. We then categorized these measures as follows: no impairment, visual impairment only, hearing impairment only, and dual sensory impairment (both visual and hearing).

### Covariates

Participants’ information concerning sociodemographic status, health behavior, and health conditions were collected by asking their age, sex, education (ie, illiterate, ≤middle school, and ≥high school), tobacco use (ever vs never), alcohol use (ever vs never), marital status (married and living together vs otherwise), residence (urban vs rural), self-reported general health status (ie, excellent, very good, good, fair, and poor), activities of daily living, and presence of certain chronic diseases. Regarding the self-reported general health status, excellent, very good, and good were grouped under good, whereas fair and poor were grouped under poor. The ADLs in CHARLS are measured using a 6-item summary assessed with an ADL scale that includes eating, dressing, transferring, bathing, using the toilet, and continence.^29^ The respondents were considered independent if they were able to complete all 6 activities without difficulty. Certain comorbidities and chronic disease conditions were captured based on self-reported chronic conditions, including hypertension, diabetes, dyslipidemia, heart problems, stroke, kidney diseases, asthma, lung diseases, arthritis, liver diseases, or stomach diseases.

### Statistical Analysis

The respondents’ characteristics were compared across categories of sensory impairments (visual impairment, hearing impairment, and dual sensory impairment) using an analysis of variance for numerical variables and ordinal χ^2^ tests for discrete variables. Continuous variables were summarized using means and SDs. Categorical variables were reported using numbers and percentages. Multiple multivariable generalized linear models (GLM) were used to assess the association of sensory impairments with cognitive function and depression. To maximize statistical power, we allowed the analytical sample size to vary depending on the number of valid responses for each cognition and depression measure. For the 3 continuous measures of cognition, we used GLM with gaussian family and identity link. Depression was measured as a dichotomous variable, so we used GLM with binomial family and log link. Age, sex, education, marital status, tobacco and alcohol use, independence (based on ADLs), place of residence (urban or rural), self-reported health status, and certain chronic conditions were included in our analytic models as covariates. Separate risk-adjusted GLM models were used to analyze the association of visual, hearing, and dual sensory impairment with cognitive function and depression. The main exposure variable was sensory impairment (categorical variable), with no sensory impairment as the reference category. We considered a 2-sided *P* < .05 statistically significant. All data analyses were conducted using Stata version 13.0 (StataCorp).

## Results

The characteristics of the respondents in the 2015 wave of the CHARLS were shown in Table 1. The study sample consisted of 18 038 respondents (9244 [51.2%] women), with a mean (SD) age of 59.9 (9.7) years. A total of 14 690 (81.4%) reported visual impairment, 11 517 (63.8%) reported hearing impairment, and 10 575 (58.6%) reported both visual and hearing impairments. Compared with respondents without visual, hearing, or dual sensory impairment, those who reported 1 or more sensory impairments had lower mean (SD) scores in memory and mental status (episodic memory score, no visual impairment vs with visual impairment: 3.6 [1.9] vs 3.3 [1.8]; mental intactness score, no hearing impairment vs with hearing impairment: 7.6 [3.1] vs 7.0 [3.2]; global cognition score among no dual sensory impairment vs with dual sensory impairment: 11.3 [4.3] vs 10.1 [4.2]). The prevalence of depression was significantly higher among respondents with at least 1 sensory impairment (eg, no visual impairment vs with visual impairment: 579 [17.3%] vs 5308 [36.2%]).

**Table 1.** Characteristics of Sample From 2015 China Health and Retirement Longitudinal Study, by Sensory Impairment Status^a^

Characteristic	Participants, No. (%)							
	Total sample (N = 18 038)	Visual impairment		Hearing impairment		Dual sensory impairment		
		Yes (n = 14 690)	No (n = 3348)	Yes (n = 11 517)	No (n = 6521)	Yes (n = 10 575)	No (n = 7463)	
Episodic memory score, mean (SD)	3.3 (1.9)	3.3 (1.8)	3.6 (1.9)	3.1 (1.8)	3.7 (1.9)	3.1 (1.8)		3.7 (1.9)
Mental intactness score, mean (SD)	7.2 (3.1)	7.1 (3.1)	7.7 (3.1)	7.0 (3.2)	7.6 (3.1)	6.9 (3.1)		7.6 (3.1)
Global cognition score, mean (SD)	10.6 (4.3)	10.4 (4.3)	11.3 (4.4)	10.1 (4.3)	11.4 (4.3)	10.1 (4.2)		11.3 (4.3)
Depression^b^	5887 (32.7)	5308 (36.2)	579 (17.3)	4474 (38.9)	1413 (21.7)	4248 (40.2)		1639 (22.0)
Age, mean (SD), y	59.9 (9.7)	60.3 (9.6)	58.1 (10.0)	61.0 (9.7)	58.0 (9.5)	61.1 (9.7)		58.3 (9.6)
Men	8794 (48.8)	7797 (53.1)	1447 (43.2)	6008 (52.2)	3236 (49.6)	5610 (53.1)		3634 (48.7)
Education								
Illiterate	4247 (23.5)	3556 (24.2)	691 (20.7)	2861 (24.9)	1386 (21.3)	2657 (25.2)		1590 (21.3)
≤Middle school	11 716 (64.9)	9541 (65.0)	2175 (65.0)	1386 (65.9)	4130 (63.4)	2657 (65.8)		4764 (63.9)
≥High school	2055 (11.4)	1576 (10.8)	479 (14.3)	1058 (9.2)	997 (15.3)	956 (9.0)		1099 (14.8)
Tobacco use								
Never	12 895 (71.5)	10 592 (72.1)	2303 68.8)	8344 (72.5)	4551 (69.8)	7681 (72.7)		5214 (69.9)
Current	5134 (28.5)	4089 (27.9)	1045 (31.2)	3167 (27.5)	1967 (30.2)	2888 (27.3)		2246 (30.1)
Alcohol use								
Never	11 548 (64.0)	9591 (65.3)	1957 (58.5)	7537 (65.5)	4011 (61.6)	6973 (66.0)		4575 (61.3)
Current	6480 (35.9)	5091 (34.7)	1389 (41.5)	3975 (34.5)	2505 (38.4)	3598 (34.0)		2882 (38.7)
Married^a^	14 793 (82.0)	12 008 (81.8)	2785 (83.2)	9390 (81.5)	5403 (82.9)	8603 (81.4)		6190 (82.9)
Rural residence	10 863 (60.2)	9022 (61.4)	1841 (55.0)	7230 (62.8)	3633 (55.7)	6683 (63.2)		4180 (56.0)
Self-reported good health status	4604 (25.5)	2785 (19.0)	1819 (54.3)	1630 (14.2)	2974 (45.6)	1293 (12.2)		3311 (44.4)
ADL impairment	3347 (18.6)	3032 (20.6)	315 (9.4)	2637 (22.9)	710 (10.9)	2492 (23.6)		855 (11.5)
Chronic diseases	13 204 (73.2)	11 133 (84.4)	2071 (75.3)	8945 (85.7)	4259 (77.3)	8294 (86.2)		4910 (77.8)
Hypertension	5524 (306)	4692 (36.7)	832 (31.6)	3764 (37.2)	1760 (33.1)	3498 (37.5)		2026 (33.3)
Diabetes	1687 (9.4)	1451 (11.5)	236 (9.1)	1187 (11.9)	500 (9.6)	1110 (12.0)		577 (9.6)
Dyslipidemia	2961 (16.4)	2519 (20.2)	442 (17.2)	1947 (19.8)	1014 (19.5)	1822 (20.1)		1139 (19.2)
Heart problems	2997 (16.6)	2599 (20.5)	398 (15.4)	2141 (21.3)	859 (16.3)	2007 (21.6)		990 (16.5)
Stroke	590 (3.2)	516 (4.1)	74 (2.9)	432 (4.3)	158 (3.0)	400 (4.3)		190 (3.2)
Kidney disease	1728 (9.6)	1519 (12.0)	209 (8.0)	1306 (13.0)	422 (8.0)	1220 (13.2)		508 (8.4)
Asthma	971 (5.3)	856 (6.7)	115 (4.4)	751 (7.5)	220 (4.2)	699 (7.5)		272 (4.5)
Chronic lung diseases	2438 (13.5)	2101 (16.5)	337 (13.0)	1799 (17.9)	639 (12.2)	1680 (18.0)		758 (12.6)
Arthritis	7158 (39.7)	6285 (49.2)	873 (33.3)	5182 (51.3)	1976 (37.3)	4870 (52.2)		2288 (37.7)
Liver disease	1155 (6.4)	995 (7.8)	160 (6.2)	834 (8.3)	321 (6.1)	768 (8.3)		387 (6.4)
Stomach disease	5203 (28.8)	4551 (35.6)	652 (24.9)	3726 (36.9)	1477 (27.9)	3484 (37.3)		1719 (28.3)

Abbreviation: ADL, activities of daily living.

^a^ Data were missing for the following characteristics: mental intactness score (270 [1.5%]), global cognition score (270 [1.5%]), depression (6 [<0.1%]), education (20 [0.1%]), tobacco use (9 [<0.1%]), alcohol use (10 [<0.1%]), marital status (1 [<0.1%]), self-reported good health status (6 [<0.1%]), chronic diseases (2097 [11.6%]), hypertension (2613 [14.5%]), diabetes (2815 [15.6%]), dyslipidemia (3021 [16.7%]), heart problems (2744 [15.2%]), stroke (2691 [14.9%]), kidney disease (2748 [15.2%]), asthma (2713 [15.0%]), chronic kidney disease (2703 [15.0%]), liver disease (2755 [15.2%]), and stomach disease (2631 [14.6%]).

^b^ Defined as a score of 10 or greater on the 10-item Center for Epidemiologic Studies–Depression scale.

**Figure 2.**
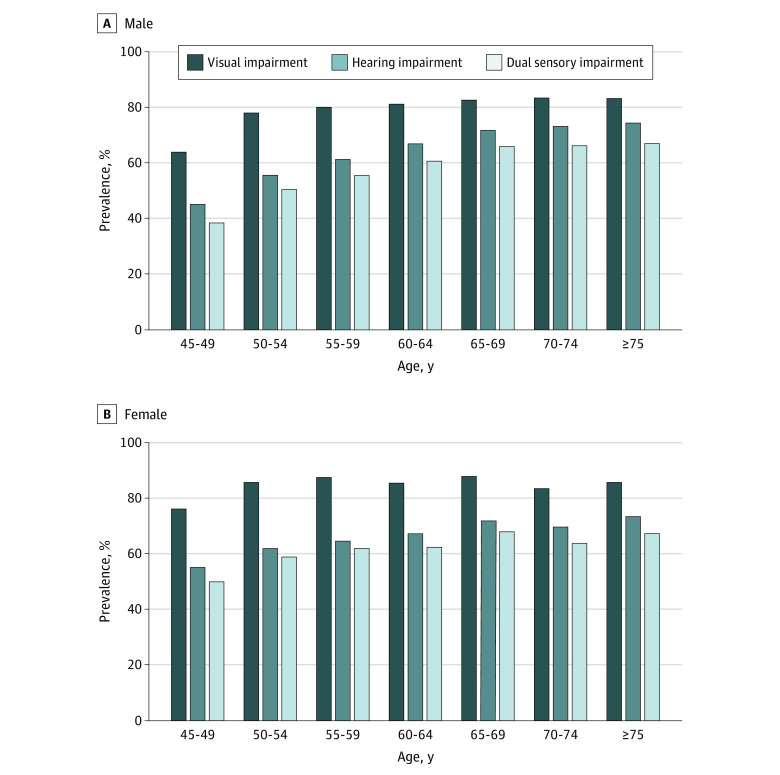
Age-Specific and Sex-Specific Prevalence of Sensory Impairments Data are from the 2015 China Health and Retirement Longitudinal Study.

Unadjusted mean rates of visual, hearing, and dual sensory impairment were clustered by age and sex (Figure 2). The direction and magnitude of sensory impairments increased by age for both men and women. Both sensory impairments were more pronounced among women compared with men in younger age categories. For example, among those younger than 70 years, women had substantially higher rates of both hearing and visual impairments than men (hearing impairment: 4920 of 7719 [63.7%] vs 4316 of 7177 [60.1%]; visual impairment: 6509 [84.3%] vs 5546 [77.3%]). These differences disappeared as people aged, with no significant differences between men and women older than age 70. Men aged between 70 and 74 years had the highest prevalence of visual impairment (681 of 819 [83.2%]), whereas those aged 75 years and older had the highest prevalence of hearing impairment (594 of 799 [74.3%]) and dual sensory impairment (484 of 721 [67.1%]). This pattern was somewhat different among women. Women aged 75 years and older reported the highest prevalence of hearing impairment (528 of 721 [73.2%]), whereas those aged 65 to 69 years reported the highest prevalence of visual impairment (1053 of 1159 [87.8%]) and dual sensory impairment (814 of 1199 [67.9%]).

**Figure 3.**
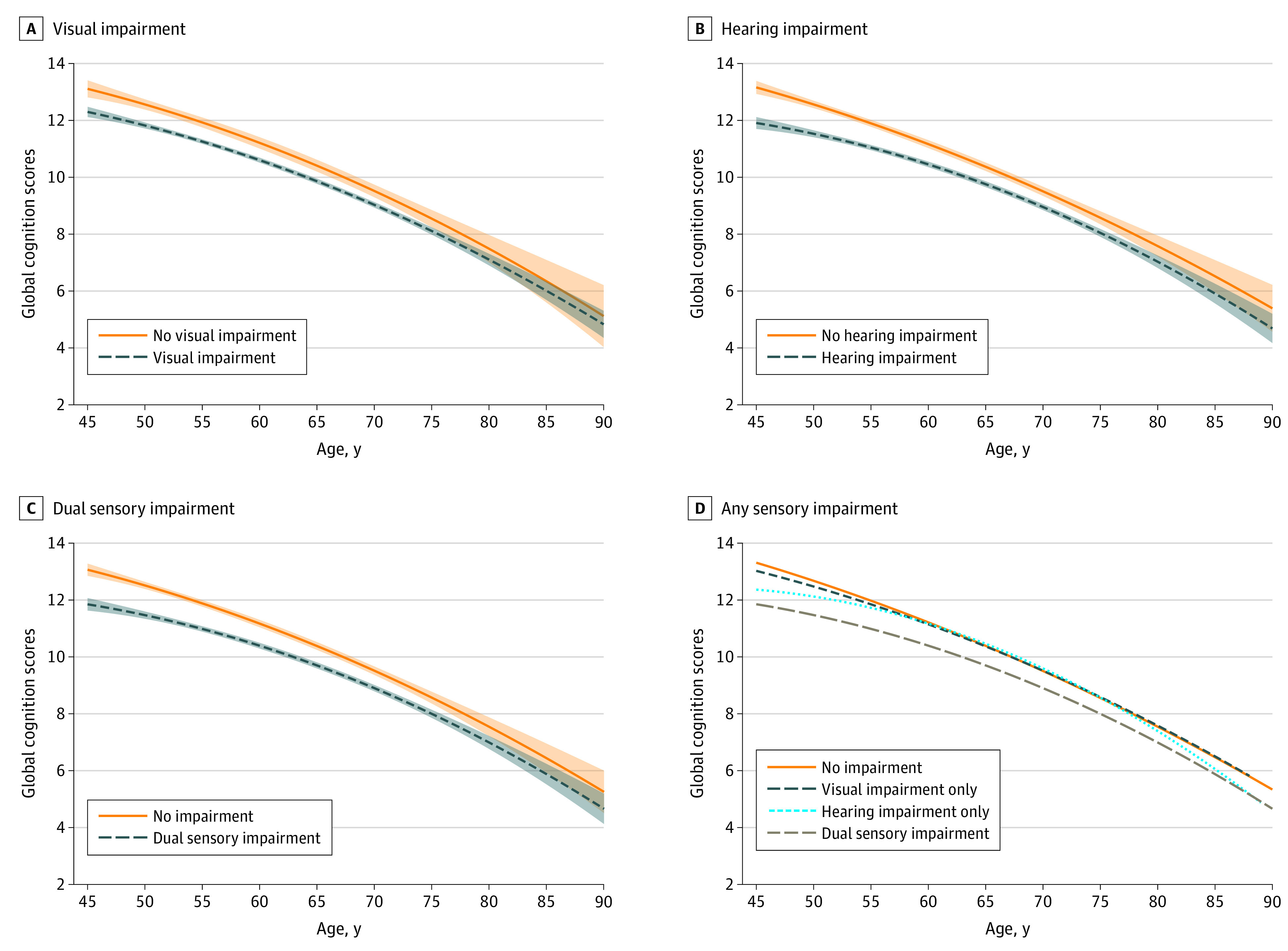
Trajectories of Cognition Scores by the Presence of Sensory Impairments in the 2015 China Health and Retirement Longitudinal Study Graphs display analog values (lines) of the global cognition score with 95% CIs (shaded areas) for visual impairment (A), hearing impairment (B), dual sensory impairment (C), and any sensory impairment (analog values only) (D).

Figure 3 shows the trajectories for cognitive function across different sensory impairment groups. We categorized sensory impairments in the simultaneous model as no impairment, visual impairment only, hearing impairment only, and dual sensory impairment. The cognitive trajectories took a downward curvilinear shape, declining with age. Visual, hearing, and dual sensory impairment were negatively correlated with the cognitive trajectory. Individuals with any sensory impairment had a substantially lower level of cognitive functioning between the ages of 45 and 75 years (mean [SD] score at 45 vs 75 years: 13.0 [3.7] vs 8.5 [4.0]). Respondents with hearing impairment showed a rapid decline in cognitive scores after the age of 75 years (mean [SD] score at age 75 years, 8.5 [4.0]; mean [SD] score per 1–age point later in life, 7.0 [4.4]).

In Table 2, after adjusting for age, sex, and other potential confounders, the presence of visual impairment was associated with decline in episodic memory (β = –0.12; 95% CI, –0.19 to –0.05) and global cognition (β = –0.16; 95% CI, –0.31 to –0.02) as well as an increase in depression (odds ratio, 1.78; 95% CI, 1.59 to 1.99). Similarly, the presence of a hearing impairment was associated with reduction in episodic memory (β = –0.24; 95% CI, –0.30 to –0.18), mental intactness (β = –0.19; 95% CI, –0.28 to –0.10), and global cognition (β = –0.43; 95% CI, –0.55 to –0.31) but an increase in depression (odds ratio, 1.57; 95% CI, 1.44 to 1.70). Furthermore, we put visual and hearing impairment in the same model and found hearing impairment associated with worse performance on all measures of cognitive function and depression, while the association between visual impairment and cognition disappeared. We further categorized sensory impairment into no impairment, visual impairment only, hearing impairment only, and dual sensory impairment. We found that respondents with visual impairment only, hearing impairment only, and dual sensory impairment were at a higher risk for depression than those without any impairment (eg, dual sensory impairment: odds ratio, 2.19; 95% CI, 1.90 to 2.52). Meanwhile, compared with respondents reporting no sensory impairment, respondents reporting dual sensory impairment scored lower in episodic memory (β = –0.23; 95% CI, –0.31 to –0.14), mental intactness (β = –0.13; 95% CI, –0.27 to –0.0003), and global cognition (β = –0.37; 95% CI, –0.55 to –0.19).

**Table 2.** Association of Sensory Impairments With Cognitive Function and Depression in Participants From the 2015 China Health and Retirement Longitudinal Study

Main independent variable^a^	β (95% CI)			Depression, OR (95% CI)^b^
	Episodic memory	Mental intactness	Global cognition	
Model 1				
Visual impairment				
No	1 [Reference]	1 [Reference]	1 [Reference]	1 [Reference]
Yes	−0.12 (−0.19 to −0.05)^c^	−0.04 (−0.15 to 0.07)	−0.16 (−0.31 to −0.02)^c^	1.78 (1.59 to 1.99)^d^
Model 2				
Hearing impairment				
No	1 [Reference]	1 [Reference]	1 [Reference]	1 [Reference]
Yes	−0.24 (−0.30 to −0.18)^d^	−0.19 (−0.28 to −0.10)^d^	−0.43 (−0.55 to −0.31)^d^	1.57 (1.44 to 1.70)^d^
Model 3				
Visual impairment				
No	1 [Reference]	1 [Reference]	1 [Reference]	1 [Reference]
Yes	−0.05 (−0.12 to 0.03)	0.03 (−0.09 to 0.14)	−0.02 (−0.18 to 0.13)	1.57 (1.40 to 1.77)^d^
Hearing impairment				
No	1 [Reference]	1 [Reference]	1 [Reference]	1 [Reference]
Yes	−0.23 (−0.29 to −0.17)^d^	−0.20 (−0.29 to −0.10)^d^	−0.43 (−0.55 to −0.30)^d^	1.44 (1.32 to 1.57)^d^
Model 4				
No sensory impairment	1 [Reference]	1 [Reference]	1 [Reference]	1 [Reference]
Visual impairment only	0.05 (−0.05 to 0.14)	0.09 (−0.05 to 0.24)	0.13 (−0.06 to 0.32)	1.50 (1.28 to 1.75)^d^
Hearing impairment only	−0.03 (−0.16 to 0.10)	−0.05 (−0.26 to 0.16)	−0.09 (−0.37 to 0.19)	1.30 (1.05 to 1.62)^c^
Dual sensory impairment	−0.23 (−0.31 to −0.14)^d^	−0.13 (−0.27 to −0.0003)^c^	−0.37 (−0.55 to −0.19)^d^	2.19 (1.90 to 2.52)^d^

Abbreviation: OR, odds ratio.

^a^ All models adjusted for age, sex, education, marital status, tobacco and alcohol use, activities of daily living, residence, self-reported general health status, and any chronic diseases.

^b^ Defined as a score of 10 or greater on the 10-item Center for Epidemiologic Studies Depression scale.

^c^ *P* < .05.

^d^ *P* < .01.

## Discussion

To our knowledge, this is the first study examining the association of sensory impairments with cognitive function and depression among adults aged 45 years or older in China. Using nationally representative data, this study had 2 major findings. First, self-reported visual, hearing, and dual sensory impairment were highly prevalent conditions among adults in China. Second, both single and dual sensory impairment were associated with poor cognitive performance and depression.

The prevalence of sensory impairments in China was much higher than in Western Europe and the United States.^30^ The Beaver Dam Offspring Study^31^ found that visual and hearing impairments among respondents aged 21 to 84 years were 14.2% and 7.8% in Beaver Dam, Wisconsin. Another cross-sectional study in Europe^32^ found even lower prevalence of visual (10.2%) and hearing (13.5%) impairments among people aged 50 years and older. The higher prevalence of sensory impairments in China may be because of the low use of eyeglasses, hearing aids, or any assistive devices.^13^ This could be explained by traditional views regarding assistive devices driven by misinformation (ie, wearing glasses can lead to further loss of eyesight^33^), financial constraints,^34^ and lack of knowledge about hearing aids.^35^ Furthermore, the differences between men and women in the prevalence of both single and dual sensory impairment were considerable, which begs for attention. In agreement with prior research, our results revealed that more women were living with sensory impairments than men.^13,36^ Social and cultural differences may expose women to a greater risk of illnesses, while social, cultural, and economic differences may reduce access to services for women.^37^

Cognition is a complex system that involves multiple domains related to episodic memory, executive function, mental intactness, and working memory.^38,39,40^ Our results indicate that respondents with single or dual impairments had poorer performance in cognitive function and more severe depression. Interestingly, when we adjusted for both visual and hearing impairment, the association between visual impairment and cognitive function disappeared, while the association between hearing impairment and cognitive function stayed intact. This might suggest that hearing impairment is more closely associated with cognitive function than visual impairment. The association between hearing impairment and cognitive decline is supported in the literature. Hearing impairment has been also linked to more severe changes in the temporal lobe structures and brain functions.^41,42^

Findings from previous research on the association between sensory impairments and cognitive decline reported conflicting results. The English Longitudinal Study of Aging found that visual and hearing impairments were associated with subsequent cognitive difficulties in older age.^43^ Similarly, a cross-sectional analysis of community-dwelling older adults in Australia revealed a negative association between visual and/or hearing impairment and Mini-Mental State Examination (MMSE) scores.^44^ In contrast, after adjusting for potential confounders, the Blue Mountains Eye Study^45^ did not find any association between visual impairment and cognition. Our findings are in agreement with studies that indicated a negative association between sensory impairment and cognitive function.^46,47,48^ For depression, our findings were in concordance with the United States National Health and Aging Study^49^ and the Korean Longitudinal Study of Aging,^50^ indicating that visual, hearing, and dual sensory impairment are associated with depression.

There are several plausible explanations for the association of sensory impairments with cognitive decline and depression. The first is the cognitive load on perception hypothesis: cognitive decline can impose a harmful cognitive load on perceptual functioning. The second is the sensory deprivation hypothesis: perceptual impairment may lead to more permanent cognitive decline because of neuroplastic changes, depression, and social isolation. The third is the information degradation hypothesis: decreased perceptual information can lead to cognitive decline among older adults. The fourth is the common cause hypothesis: a common cause results in both age-related cognitive decline and sensory impairments in the process of neural degeneration.

Our findings agree with the sensory impairment deprivation hypothesis and the common cause hypothesis. The respondents with sensory impairments in the present study had a worse ADL functional status and more severe depression. Previous studies^13,51^ revealed that people with sensory impairment had lower ADLs and experienced more depression and social isolation. The sensory impairment deprivation hypothesis indicates that depression and social isolation may mediate the association between sensory impairments and cognitive decline.^52,53^ Research suggests that visual impairment and dual sensory impairment are associated with depression because of increased social isolation and loneliness.^54,55^ Less frequent social interactions may result in cognitive decline in older age.^56^ The common cause hypothesis, which states that sensory impairments as well as cognitive decline and depression may be caused by a common pathological process, such as vascular disease, should also be considered. For example, β-amyloid pathology might damage both sensory and cognitive abilities.^57^ These hypotheses are not mutually exclusive, and the present cross-sectional study does not allow for a conclusive distinction between them. More studies are needed to further investigate these hypotheses.

### Limitations

This study has limitations. First, visual and hearing sensations were assessed though self-reported measures, without any comparative objective sensory data. Second, the cross-sectional nature of the design meant we could determine the mechanistic basis of the observed association of sensory impairments with cognitive decline and depressive symptoms, so the findings should be interpreted with caution. Third, the study did not investigate whether assistive devices (eg, hearing aids, glasses, or portable magnifiers) could potentially mitigate cognitive function and depressive symptoms.

## Conclusions

In this study, the association of sensory impairments with cognitive function and depression were explored using a nationally representative sample in China. The findings suggest that sensory impairments are associated with a higher risk of cognitive decline and depression. The association between hearing impairment and cognitive function among older adults is of paramount importance. There is a dearth of epidemiological studies on this topic in China. Considering China’s rapidly aging population and high prevalence of sensory impairment, this is a salient topic with important clinical and public health implications.
